# The patient journeys of children and adolescents with depression: a study of electronic health records

**DOI:** 10.1007/s00787-023-02232-6

**Published:** 2023-05-25

**Authors:** Alice Wickersham, Juliette Westbrook, Craig Colling, Johnny Downs, Risha Govind, Daisy Kornblum, Jonathan Lewis, Patrick Smith, Tamsin Ford

**Affiliations:** 1 CAMHS Digital Lab, Department of Child and Adolescent Psychiatry, King’s College London, London, UK; 2 Department of Psychology, King’s College London, London, UK; 3 South London and Maudsley Mental Health NHS Trust, London, UK; 4 Institute of Psychiatry, Psychology and Neuroscience, King’s College London, London, UK; 5 Cambridgeshire and Peterborough NHS Foundation Trust, Cambridge, UK; 6 Department of Psychiatry, University of Cambridge, Cambridge, UK

**Keywords:** Depression, Children, Adolescence, Mental Health Services

## Abstract

In England, children and adolescents with depression can seek treatment from specialist mental health services. We know little about how they journey through these services, or whether healthcare providers collect sufficient data to accurately appraise this. We aimed to summarise the child and adolescent depression pathway for two healthcare providers. This cohort study used de-identified electronic health records extracted from Cambridgeshire and Peterborough NHS Foundation Trust (CPFT) and South London and Maudsley NHS Foundation Trust (SLaM). We identified referrals between 2015 and 2019 during which the referred patient received their first depression diagnosis aged < 18 years. We described patient demographic and clinical characteristics, and features of the referral. In total, *n* = 296 (CPFT) and *n* = 2502 (SLaM) patients had a referral which met eligibility criteria. In both sites, patients were more frequently female (CPFT 79.3%; SLaM 69.3%) and White ethnicity (CPFT 88.9%; SLaM 57.9%) as compared to respective population estimates for the Trusts’ catchment areas. Patients typically received their first depression diagnosis during adolescence (median ages 16 in CPFT and 15 in SLaM). The most common comorbidity was anxiety disorder. Referrals were usually routine, to community teams specialising in the child age group. Commonly mentioned interventions included antidepressant medication, cognitive behavioural therapy, and dialectical behaviour therapy. However, pathways varied within and between sites, and the quality and consistency of some data was poor. These findings provide an overview of service pathways experienced by children and adolescents with depression, but also highlight that pathways can vary according to individual need and healthcare provider. More systematic collection of some data, and standardisation in record systems used by different providers, would be beneficial.

**Supplementary Information:**

The online version contains supplementary material available at 10.1007/s00787-023-02232-6.

## Introduction

Depression is a leading cause of illness and disability among adolescents worldwide [1], and is characterised by low mood, irritability, low self-esteem and suicidal ideation [2]. The prevalence of depression increases steeply with age, from 0.3% during primary school (ages 5 to 10 years) to 2.7% during secondary school (ages 11 to 16 years) according to English estimates [3].

Contact with mental health services significantly reduces depression symptoms in adolescents [4]. In England, evidence-based depression treatment is available through Child and Adolescent Mental Health Services (CAMHS), and may include medication and psychological therapy [5]. Statistics about mental health services are submitted to, and reported by, NHS Digital’s Mental Health Services Data Set (MHSDS) [6]. But currently, little detailed information is reported on the referral, treatment, and discharge pathway, specifically for children and adolescents with depression who have been referred and accepted to CAMHS.

Having a clear picture of how child and adolescent depression is treated by mental health services is critical for understanding whether services are operating as effectively as possible and in accordance with treatment guidelines. This is important for benchmarking and for commissioning on local and national levels. However, it is also unclear what administrative data on the child and adolescent depression pathway are systematically available at the NHS Trust-level, or their quality.

We therefore sought to provide an overview of the mental health service pathway accessed by children and adolescents with depression in two NHS Trusts, and to determine the current availability and quality of data available to appraise these pathways.

## Methods

### Design and sample

This historical cohort study is reported according to RECORD guidelines (Supplement 1) [7]. We used data from two healthcare providers in England: Cambridgeshire and Peterborough NHS Foundation Trust (CPFT), and South London and Maudsley NHS Foundation Trust (SLaM). CPFT provides mental health care to an East England catchment area, while SLaM provides secondary mental health services to four south London boroughs (Croydon, Lambeth, Lewisham and Southwark), as well as some national and specialist outpatient services which are also accessible to patients from outside the catchment area. The catchment areas have quite different sociodemographic profiles: compared to CPFT, SLaM’s catchment has greater ethnic diversity, with a lower proportion of individuals from White ethnic backgrounds, and also a higher proportion of deprived areas (Table 1).

**Table 1:** Socio-demography of CPFT and SLaM catchment areas, as compared to England

	CPFT^a^	SLaM^b^	England
Population (2021)	896,756	1,314,188	56,536,419
Sex (2021)			
Male	49.4%	48.1%	49.0%
Female	50.6%	51.9%	51.0%
Age (years) (2021)			
< 18	20.9%	20.2%	20.8%
≥ 18	79.1%	79.8%	79.2%
Ethnicity (2021)			
White	85.4%	51.4%	81.7%
Black, black British, Caribbean or African	2.1%	24.5%	4.0%
Asian or Asian British	7.9%	11.3%	9.3%
Mixed or multiple ethnic groups	3.0%	7.7%	2.9%
Other ethnic group	1.7%	5.1%	2.1%
Proportion of LSOAs in each IDACI quintile (2019)			
1 (most deprived)	10.1%	38.4%	20.0%
2	20.1%	34.1%	20.0%
3	20.9%	17.1%	20.0%
4	24.0%	8.7%	20.0%
5 (least deprived)	24.8%	5.3%	20.0%

*IDACI,* Income Deprivation Affecting Children Index; *LSOA,* Lower Super Output Area. Estimates are derived from UK government and Office for National Statistics publicly available data sources [8–10]

^a^Includes Peterborough, Fenland, Huntingdonshire, East Cambridgeshire, South Cambridgeshire, Cambridge

^b^Includes Croydon, Lambeth, Lewisham and Southwark

We analysed data from de-identified electronic health records. In CPFT, data were extracted and pseudonymised from the RiO electronic health record system using Clinical Records Anonymisation and Text Extraction (CRATE) [11]. In SLaM, data were extracted and pseudonymised from the electronic Patient Journey System via the Clinical Record Interactive Search (CRIS) [12].

We identified referrals to each site which overlapped with the 5-year period 2015 to 2019, and during which the referred patient received their *first* depression diagnosis, among children and young people who were under 18 years of age. We defined depression diagnosis using structured diagnosis fields as an F32.x or F33.x code (10th Revision of the World Health Organization International Statistical Classification of Diseases and Related Health Problems, ICD-10) [13]. We excluded ‘rejected’ referrals (SLaM), ‘inappropriate referrals’ (CPFT), and referrals recorded as taking place at age 0 years. In CPFT, if patients had multiple referrals or episodes that met eligibility criteria, we ordered referrals based on referral and discharge date, and took the first such instance across these two variables. In SLaM, we ordered referrals based on referral date, acceptance date and episode ID, and kept the first such instance across all three variables. As a result, the referral under study may not always be with the team who made the depression diagnosis, but will still give an overview of one of the concurrent treatment pathways being accessed by the patient at that time. A total *n* = 296 were eligible for inclusion in CPFT, and *n* = 2502 in SLaM.

We derived variables from structured fields (more detail below), but for some referrals in CPFT only, we also conducted a manual audit of de-identified free-text clinical notes. This audit was conducted to populate variables which could not be derived for CPFT using structured fields or natural language processing (NLP) (in SLaM, all variables could be derived using these methods, and so a manual audit was not required). For the manual audit of CPFT notes, we focussed on a subset of *n* = 40 closed referrals (i.e. those who had been discharged) which were recorded under the ‘child’ speciality, ‘community’ area, and a ‘general psychiatry’ team. Because the study was undertaken during a limited timeframe, *n* = 40 was as many records as the lead researcher was able to audit in the time allotted. We sampled these referrals so that they represented patients who were with the service for a range of durations (from < 6 months to ≥ 18 months).

### Ethics and consent

The CPFT Research Database was approved by the NHS East of England – Cambridge Central Research Ethics Committee (references 12/EE/0407, 17/EE/0442), and aspects of the study described here relating to CPFT were approved by their Research Database Oversight Committee. CRIS has received research ethics committee approval as a database for secondary analysis (Oxford REC C reference 18/SC/0372), and aspects of the study described here relating to SLaM were approved by the CRIS Oversight Committee. Both databases operate on an opt-out basis.

### Measures

#### Demographic and clinical characteristics

Gender was extracted from structured fields. Ethnicity was extracted from structured fields and categorised as White/Black/Asian/Mixed/Any other ethnic group. We calculated age at referral and depression diagnosis from the date of birth, referral date and diagnosis date.

Comorbidities were extracted from structured diagnosis fields. We summarised all diagnoses made at any time before the end of the referral period. Therefore, some of the comorbidities reported may have been identified before the referral in question, and it is possible that the patients no longer met criteria for these comorbidities at the time of first depression diagnosis. We focussed on ICD-10 codes indicative of anxiety disorders (F40.x-F48.x), eating disorders (F50.x), personality disorders (F60.x and F61), learning/developmental disorders (including autism) (F70-F79 and F80.x-F89), hyperkinetic disorders (F90.x), and psychotic disorders (F20.x-F29).

#### Service pathway characteristics

Service pathway characteristics were summarised from structured fields, including referral source, urgency, destination speciality (child or adult / other) and area (community or inpatient). For referrals under the child speciality and to community areas, we also summarised the team type. We inferred whether the referral was still open at the time of data extraction based on whether the discharge date field was populated. If the referral was closed, we also summarised discharge reason. For some variables, we combined categories to ensure that cell sizes were not disclosive–detail is provided in Supplement 2.

#### Interventions

We used NLP applications to extract mentions of medications from clinical notes, and inspected the resulting variable for psychotropic medications. We focussed on mentions of psychotropic medications where the first mention of the medication was during the referral period (i.e. after the referral date, and if the referral had been closed, before the discharge date). These were categorised into antidepressants, antipsychotics, mood stabilisers, and sleeping pills/tranquilisers (Supplement 3).

In SLaM, we used NLP to extract mentions of therapeutic interventions. Again, we focussed on courses of therapy where the start date fell within the referral period. If patients started multiple courses of therapy within the referral period, we focussed on the first course of therapy, describing type of therapy and number of sessions attended. In CPFT, therapeutic modality used as a first-line intervention was extracted during the manual audit of de-identified free-text clinical notes (and therefore limited to the sample of closed referrals under the ‘child’ speciality, ‘community’ area, and a ‘general psychiatry’ team).

#### Service pathway timings

In SLaM, we calculated time to first contact for referrals made under the ‘child’ speciality and to ‘community’ areas. Referral date was extracted from a structured field, and first contact date also extracted from a structured field containing the date when the patient first attended a face-to-face or remote session under the team.

In CPFT, time to assessment was ascertained during the manual audit of free-text clinical notes (and therefore limited to the sample of closed referrals under the ‘child’ speciality, ‘community’ area, and a ‘general psychiatry’ team). Referral date was extracted from a structured field, and assessment date ascertained from the free-text. Sometimes assessment dates were censored in the clinical notes. In these cases, the assessment date was taken to be the date that assessment notes were seemingly uploaded to the patient record, although this could have been done some time after the assessment took place.

For referrals which had been discharged by the time of data extraction for this study, total time of referral was calculated as time from referral date to discharge date, which in both sites was extracted from structured fields.

### Statistical analysis

We descriptively summarised the variables using frequencies and percentages (categorical variables) or medians and interquartile ranges (continuous variables) to understand aspects of the depression treatment pathway. Analyses were carried out in R versions 4.0.3 (CPFT) and 4.2.0 (SLaM).

## Results

### Demographic and clinical characteristics

In total, *n* = 296 (CPFT) and *n* = 2502 (SLaM) patients had a referral which met eligibility criteria. In both sites, patients were mostly female and White ethnicity (Table 2). Compared to the population of each site’s catchment area, the proportion of patients who were female and White ethnicity was relatively high (Table 1). Typically, referral and first depression diagnosis took place in late adolescence (Table 2). Both sites showed an increasing gradient towards higher ages at first depression diagnosis, and this was particularly the case in CPFT, where the median age at first depression diagnosis (16.0 years) was slightly higher than in SLaM (15.0 years) (Table 2). In both sites, approximately half the sample had comorbidities, the most common of which was anxiety disorder.

**Table 2:** Patient demographic and clinical characteristics

Demographic and clinical characteristics	CPFT (*n* = 296)		SLaM (*n* = 2502)	
	*n*	%	*n*	%
Gender				
Female	234	79.3	1732	69.3
Male	61	20.7	749	30.0
Other	N/A	N/A	17	0.7
Ethnicity				
White	240	88.9	1283	57.9
Black	< 10	^a^	458	20.7
Asian	< 10	^a^	146	6.6
Mixed	13	4.8	212	9.6
Any other ethnic group	< 10	^a^	116	5.2
Age at first depression diagnosis				
< 11	< 10	^a^	73	2.9
11	< 10	^a^	72	2.9
12	< 10	^a^	111	4.4
13	16	5.4	222	8.9
14	34	11.5	348	13.9
15	59	19.9	465	18.6
16	68	23.0	547	21.9
17	115	38.9	664	26.5
Median (interquartile range)	16.0 (15.0 to 17.0)		15.0 (14.0 to 17.0)	
Age at referral for episode of care in which depression first diagnosed				
< 11	< 10	^a^	116	4.6
11	< 10	^a^	78	3.1
12	18	6.1	131	5.2
13	44	14.9	276	11.0
14	48	16.2	416	16.6
15	49	16.6	492	19.7
16	42	14.2	522	20.9
17	86	29.1	471	18.8
Median (interquartile range)	15.0 (14.0 to 17.0)		15.0 (14.0 to 16.0)	
Comorbidities				
Anxiety disorder	95	32.1	749	29.9
Eating disorder	60	20.3	168	6.7
Personality disorder	24	8.1	77	3.1
Learning/developmental disorder (including autism)	20	6.8	297	11.9
Hyperkinetic disorder	15	5.1	121	4.8
Psychotic disorder	< 10	^a^	23	0.9
Any of the above	171	57.8	1126	45.0

In CPFT, gender was missing for *n* = 1; ethnicity was missing for *n* = 26. In SLaM, gender was missing for *n* = 4; ethnicity was missing for *n* = 287. *N/A*, category does not apply to this site

^a^Suppressed to avoid potentially disclosive cell sizes

### Service pathway characteristics

Most referrals were made from primary care, and were routine rather than urgent/priority (Table 3). Most were made under the ‘child’ speciality (rather than adult/other), and to ‘community’ areas (rather than inpatient). ‘Child’ and ‘community’ referrals were mostly made to general mental health teams, although some were referred to more specialist teams (such as for eating disorders) (Table 4).

**Table 3:** Service pathway characteristics

Service pathway characteristics	CPFT (*n* = 296)		SLaM (*n* = 2502)	
	*n*	%	*n*	%
Referral source				
Primary care	172	58.1	772	32.1
A&E/crisis team/first response	24	8.1	432	18.0
Education setting	< 10	^a^	164	6.8
Justice/forensic setting	< 10	^a^	11	0.5
Self or carer referral	< 10	^a^	50	2.1
Family or care services	< 10	^a^	72	3.0
Other (including other mental health services)	88	29.7	901	37.5
Referral urgency				
Routine	223	75.3	2109	84.8
Urgent/priority	73	24.7	379	15.2
Speciality				
Child	217	73.3	2352	94.0
Adult/other	79	26.7	150	6.0
Area description				
Community	253	85.5	2386	95.4
Inpatient	43	14.5	116	4.6
Medication				
Antidepressants	257	86.8	1069	42.7
Antipsychotics	91	30.7	301	12.0
Sleeping pills/tranquilisers	156	52.7	628	25.1
Mood stabilisers	13	4.4	56	2.2
Any of the above	267	90.2	1184	47.3
Referral still open at time of extraction				
No	255	86.1	2463	98.4
Yes	41	13.9	39	1.6
Discharge reason (closed referrals only)				
Care ended	166	65.1	1950	79.2
Care from elsewhere	17	6.7	357	14.5
Withdrawal or death	30	11.8	156	6.3
Other	42	16.5	N/A	N/A

In SLaM, data was missing for *n* = 100 on referral source, *n* = 14 on referral urgency. *A&E,*Accident & Emergency; *N/A,* category does not apply to this site. ^a^Suppressed to avoid potentially disclosive cell sizes

**Table 4:** 'Child’ and ‘community’ referrals destination team type

Team type	SLaM (*n* = 2236)	
	*n*	%
General community CAMHS	930	41.6
Eating disorder or weight	218	9.7
Neurological	200	8.9
Early intervention	166	7.4
Liaison psychiatry	143	6.4
Emergency or crisis team	109	4.9
Anxiety, mood or trauma	91	4.1
Looked after children	85	3.8
Forensic or offending	37	1.7
Other	257	11.5

	CPFT (*n* = 175)	
	*n*	%
General psychiatry	153	87.4
Community eating disorder service	12	6.9
Autism spectrum disorder service	< 10	^a^
Crisis resolution team / home treatment	< 10	^a^
Learning disability service	< 10	^a^
Substance misuse team	< 10	^a^
Other mental health service	< 10	^a^

*CAMHS, *Child and Adolescent Mental Health Services. ^a^Suppressed to avoid potentially disclosive cell sizes

In both sites, most referrals were closed by the time of data extraction for this study (slightly more in SLaM, likely because data extraction was conducted later than in CPFT) (Table 3). Among the discharged referrals, the predominant reason for discharge was because care ended (e.g. due to treatment completion, or on professional advice).

### Interventions

There were more mentions of psychotropic medications in CPFT than in SLaM (Table 3). However, in both sites, the most commonly mentioned medications were antidepressants, followed by sleeping pills / tranquilisers, antipsychotics, then mood stabilisers.

In SLaM, of the *n* = 86 referrals where a type of therapy was mentioned, the most common therapy was cognitive behavioural therapy (CBT) or dialectical behavioural therapy (DBT), followed by art therapies and group therapy (Table 5). The median number of sessions attended varied slightly by therapy type.

**Table 5:** Therapy type and sessions attended, SLaM (*n* = 86)

Therapy type	*n*	%	Number of sessions attended (median, interquartile range)
CBT or DBT	35	40.7	11.0 (6.0 to 14.5)
Arts therapies	15	17.4	13.0 (5.0 to 20.0)
Group therapy	14	16.3	11.0 (6.0 to 14.8)
Other	22	25.6	11.0 (6.5 to 23.8)

In CPFT, therapeutic interventions were inconsistently recorded in free-text clinical notes. In the majority of cases, it was difficult to infer what therapeutic modality was offered as a first line. This was particularly the case for longer or more complex referrals, or referrals where the patient was seen by multiple agencies or received inpatient care; in these instances it was difficult to infer which of many different approaches taken could be considered the first-line treatment for depression. Of the *n* = 40 whose free-text clinical notes were manually audited, *n* = 14 seemingly underwent CBT or DBT as one of the first lines of therapeutic intervention, and *n* < 10 made reference to other therapeutic modalities. The remaining manually audited clinical notes either made no reference to therapy, did not specify therapeutic modality, or were unclear as to whether the patient underwent the therapy.

### Service pathway timelines

In SLaM, time to first contact was available for *n* = 2139 referrals. Most were seen within 1 month (*n* = 1043, 48.8%), followed by 1–6 months (*n* = 947, 44.3%), and 7–12 months (*n* = 114, 5.3%); few waited more than 12 months (*n* = 35, 1.6%).

In CPFT, an assessment date could only be identified for *n* = 24 of the *n* = 40 referrals whose free-text clinical notes were manually audited. Of these, *n* = 10 (41.7%) were assessed less than 1 month after referral. The remaining *n* = 14 (58.3%) were assessed 1–6 months after referral.

**Fig. 1:**
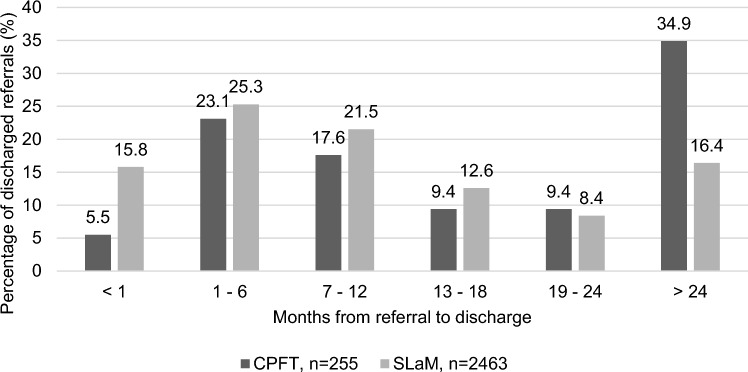
Months from referral to discharge

Among the referrals who were discharged by the time of data extraction for this study, the total time in months from referral to discharge varied widely (Fig. 1). Both sites suggested a pattern where patients who had not been discharged within 1 year of referral often remained with the team or service for over 2 years.

## Discussion

This study provides an overview of mental health service pathways experienced by children and adolescents with depression across two NHS trusts serving a range of populations, from highly ethnically diverse metropolitan areas to rural and semirural areas. Consistent with previous findings among those seeking help for depression in this age group [2], patients were mostly female, depression was usually identified during mid- to late-adolescence, and comorbid anxiety was common. Referrals were usually routine, to community teams specialising in the child age group. Many had their first appointment or assessment within 1 or 6 months of referral, but total time to discharge varied widely. Consistent with NICE guidelines, interventions commonly employed by both sites were antidepressant medications, CBT, and DBT [5]. However, there was a great deal of variation in the service pathways experienced by children and adolescents with depression, likely due to differences in their individual needs. It is concerning that between a third and half of these referrals were in contact with services for more than a year, and future research could address what predicts such long service episodes and what might support young people to recover more quickly.

There was also some variation between the two sites, which is expected: compared to CPFT, SLaM serves a more diverse and deprived population, which will increase the level of need, and offers national and specialist outpatient services, such that the needs of individuals accessing SLaM may tend towards the more severe or complex. An important implication of this variation between the sites is that studies which sample from a particular healthcare provider or region should fully describe the characteristics and services underlying their population so the reader can appraise generalisability to other areas. This is particularly pertinent for clinical trials that employ a ‘treatment as usual’ group. Our findings highlight that ‘treatment as usual’ can vary substantially between patients and healthcare providers, and without investigating this thoroughly, we are left unclear as to what the intervention under study is being compared to. Indeed, some ‘treatment as usual’ groups may comprise individuals who are receiving care which is adequate, timely, comprehensive, and well-suited to their needs, a phenomenon which is thought to be contributing to increasingly small effect sizes in clinical trials [14].

The purpose of this study was to describe the service pathways in the two sites, not to directly compare them. While we replicated our methods as closely as possible between the sites, much of the variation observed between CPFT and SLaM may be attributable to differences in how data are recorded, extracted, processed and categorised. The two sites use different electronic health record systems, and different data extraction tools. This itself highlights a challenge for researchers using electronic health records to understand regional variations in mental health and healthcare: the extent to which data from different sites are directly comparable is questionable, and researchers attempting to draw such comparisons need to consult closely with clinicians and informaticians from each site to understand what information is being recorded, and how.

Databases like MHSDS are important for giving broad mental health service statistics and benchmarking [6]. However, the variations we observed in recording systems, and the difficulties we encountered in reliably ascertaining some information like time to first appointment or assessment, highlights that the data provenance for such databases require further scrutiny. The need to improve data quality has also been noted by other work using routinely collected health data to investigate child and adolescent mental health services [15]. Greater standardisation in the record systems used by different providers would be beneficial, as would more systematic collection of some data. For example, quality and consistency of routine outcome measurement is currently poor [16], although efforts are being made to improve this [17].

Some limitations of this study should be highlighted. The findings are limited to two NHS Trusts in England, chosen because these were the two sites which the lead researcher had access to while undertaking a placement, and because they have CRIS and CRATE systems with robust governance frameworks for conducting health services research using de-identified CAMHS records. The findings may therefore not be generalisable to other areas. Indeed, as discussed, the differences we observed between CPFT and SLaM suggest that service pathways will likely be very different again elsewhere, due to variation in the services offered and record systems used.

We did not conduct validation work on the variables, and so are unable to estimate the extent of recording error and bias. For example, there is known to be a degree of diagnostic and administrative error in structured diagnosis fields [18]. Structured fields are also necessarily limited in the amount of nuance and detail they can capture; interviews with clinicians, patients, and audits of free-text clinical notes may be beneficial for further understanding the complexities of accessing mental health services for depression.

We cannot ascertain which of the referrals and treatments we captured were primarily for depression. For some, depression might be a secondary diagnosis, and may not be currently actively treated. For example, the referral under study might instead be targeting an eating disorder or neurodevelopmental disorder. Nonetheless, capturing these referrals is still informative for understanding interactions between children and adolescents with depression and healthcare providers, and for highlighting the extent of variation in their individual needs.

Finally, in this study we only describe service pathways for help-seeking children and adolescents who were referred to, and accepted by, secondary mental healthcare services. Some patients may have their referrals to CAMHS services rejected because they do not meet certain thresholds for severity or complexity. In this study we were unable to investigate referrals for depression which were not accepted, because a depression diagnosis is usually only registered in CAMHS electronic health records after the referral has been accepted and the patient assessed—therefore, we cannot confidently or accurately ascertain which rejected referrals were specifically made for depression. Additionally, many in the general population with depression do not seek support, seek informal support from family or teachers, or receive care from private or third sector organisations [19]. Understanding alternative service pathways for children and adolescents with depression that involve third sector and private providers is an important area for future work.

In conclusion, while many aspects of the mental health service pathways we describe are as expected for children and adolescents with a depression diagnosis, we observed a great deal of variation between individuals and sites. Some of this variation will reflect genuine differences in individual needs and services offered, highlighting that depressive disorders and approaches to treatment are highly heterogenous. However, some variation could also reflect differences in how data are recorded within and between sites, highlighting a need for improved and standardised data collection to fully appraise healthcare provision and associated regional differences.

### Supplementary Information

Below is the link to the electronic supplementary material.

Supplementary file1 (PDF 355 KB)

## Data Availability

The data cannot be made publicly available, but can be accessed with permissions from the South London and Maudsley NHS Foundation Trust and Cambridgeshire and Peterborough NHS Foundation Trust. RG (SLaM), DK (SLaM) and JL (CPFT) had access to the database populations used to create the study population, and extracted the study data. At the time of submission, AW has full and ongoing access to the extracted study data.

## Code availability

R code used for data analysis will be uploaded to the corresponding author’s GitHub upon publication (AW, https://github.com/AliceWickersham).
