# Supplementary material for: The patient journeys of children and adolescents with depression: a study of electronic health records
Source: Eur Child Adolesc Psychiatry. 2023 May 25;33(4):1093–101. doi: 10.1007/s00787-023-02232-6 (PMC11032266; doi:10.1007/s00787-023-02232-6)
Supplement: Supplementary file 1 — Supplementary file1 (PDF 355 KB) [file 787_2023_2232_MOESM1_ESM.pdf]

Supplementary information for "The patient journeys of children and adolescents with depression: A study of electronic health records", Wickersham, A., Westbrook, J., Colling, C., Downs, J., Govind, R., Kornblum, D., Lewis, J., Smith, P., Ford, T.

Corresponding author: Dr Alice Wickersham, CAMHS Digital Lab, King's College London, London, UK, alice.wickersham@kcl.ac.uk

Supplement 1: The RECORD statement – checklist of items, extended from the STROBE statement, that should be reported in observational studies using routinely collected health data. Items specific to cross-sectional/case-control studies have been removed.

	Item No.	STROBE items	Page	RECORD items	Page
**Title and abstract**					
	1	(a) Indicate the study's design with a commonly used term in the title or the abstract (b) Provide in the abstract an informative and balanced summary of what was done and what was found	3	RECORD 1.1: The type of data used should be specified in the title or abstract. When possible, the name of the databases used should be included.<br><br>RECORD 1.2: If applicable, the geographic region and timeframe within which the study took place should be reported in the title or abstract.<br><br>RECORD 1.3: If linkage between databases was conducted for the study, this should be clearly stated in the title or abstract.	3
**Introduction**					
Background rationale	2	Explain the scientific background and rationale for the investigation being reported	4	N/A	
Objectives	3	State specific objectives, including any prespecified hypotheses	4	N/A	
**Methods**					
Study Design	4	Present key elements of study design early in the paper	5	N/A	
Setting	5	Describe the setting, locations, and relevant dates, including periods of recruitment, exposure, follow-up, and data collection	5-6	N/A	
Participants	6	*(a) Cohort study* - Give the eligibility criteria, and the sources and methods of selection of participants. Describe methods of follow-up	5-6	RECORD 6.1: The methods of study population selection (such as codes or algorithms used to identify subjects)	5-6

	Item No.	STROBE items	Page	RECORD items	Page
		(b) Cohort study - For matched studies, give matching criteria and number of exposed and unexposed		should be listed in detail. If this is not possible, an explanation should be provided.<br><br>RECORD 6.2: Any validation studies of the codes or algorithms used to select the population should be referenced. If validation was conducted for this study and not published elsewhere, detailed methods and results should be provided.<br><br>RECORD 6.3: If the study involved linkage of databases, consider use of a flow diagram or other graphical display to demonstrate the data linkage process, including the number of individuals with linked data at each stage.	
Variables	7	Clearly define all outcomes, exposures, predictors, potential confounders, and effect modifiers. Give diagnostic criteria, if applicable.	6-7	RECORD 7.1: A complete list of codes and algorithms used to classify exposures, outcomes, confounders, and effect modifiers should be provided. If these cannot be reported, an explanation should be provided.	6-7
Data sources/ measurement	8	For each variable of interest, give sources of data and details of methods of assessment (measurement).<br>Describe comparability of assessment methods if there is more than one group	6-7	N/A	
Bias	9	Describe any efforts to address potential sources of bias	N/A	N/A	
Study size	10	Explain how the study size was arrived at	5-6	N/A	
Quantitative variables	11	Explain how quantitative variables were handled in the analyses. If applicable, describe which groupings were chosen, and why	6-7	N/A	
Statistical methods	12	(a) Describe all statistical methods, including those used to control for confounding<br>(b) Describe any methods used to examine subgroups and interactions<br>(c) Explain how missing data were addressed	7	N/A	

	Item No.	STROBE items	Page	RECORD items	Page
		(d) *Cohort study* - If applicable, explain how loss to follow-up was addressed<br>(e) Describe any sensitivity analyses			
Data access and cleaning methods		N/A		RECORD 12.1: Authors should describe the extent to which the investigators had access to the database population used to create the study population.<br><br>RECORD 12.2: Authors should provide information on the data cleaning methods used in the study.	2
Linkage		N/A		RECORD 12.3: State whether the study included person-level, institutional-level, or other data linkage across two or more databases. The methods of linkage and methods of linkage quality evaluation should be provided.	N/A
**Results**					
Participants	13	(a) Report the numbers of individuals at each stage of the study (*e.g.*, numbers potentially eligible, examined for eligibility, confirmed eligible, included in the study, completing follow-up, and analysed)<br>(b) Give reasons for non-participation at each stage.<br>(c) Consider use of a flow diagram	N/A	RECORD 13.1: Describe in detail the selection of the persons included in the study (*i.e.,* study population selection) including filtering based on data quality, data availability and linkage. The selection of included persons can be described in the text and/or by means of the study flow diagram.	5-6
Descriptive data	14	(a) Give characteristics of study participants (*e.g.*, demographic, clinical, social) and information on exposures and potential confounders<br>(b) Indicate the number of participants with missing data for each variable of interest<br>(c) *Cohort study* - summarise follow-up time (*e.g.*, average and total amount)	8-9	N/A	
Outcome data	15	*Cohort study* - Report numbers of outcome events or summary measures over time	10-12	N/A	
Main results	16	(a) Give unadjusted estimates and, if applicable, confounder-adjusted estimates and their precision (e.g., 95% confidence	N/A	N/A	

	Item No.	STROBE items	Page	RECORD items	Page
		interval). Make clear which confounders were adjusted for and why they were included<br>(b) Report category boundaries when continuous variables were categorized<br>(c) If relevant, consider translating estimates of relative risk into absolute risk for a meaningful time period			
Other analyses	17	Report other analyses done—e.g., analyses of subgroups and interactions, and sensitivity analyses	N/A	N/A	
**Discussion**					
Key results	18	Summarise key results with reference to study objectives	13	N/A	
Limitations	19	Discuss limitations of the study, taking into account sources of potential bias or imprecision. Discuss both direction and magnitude of any potential bias	13-14	RECORD 19.1: Discuss the implications of using data that were not created or collected to answer the specific research question(s). Include discussion of misclassification bias, unmeasured confounding, missing data, and changing eligibility over time, as they pertain to the study being reported.	13-14
Interpretation	20	Give a cautious overall interpretation of results considering objectives, limitations, multiplicity of analyses, results from similar studies, and other relevant evidence	13-14	N/A	
Generalisability	21	Discuss the generalisability (external validity) of the study results	13	N/A	
**Other Information**					
Funding	22	Give the source of funding and the role of the funders for the present study and, if applicable, for the original study on which the present article is based	2	N/A	
Accessibility of protocol, raw data, and programming code		N/A		RECORD 22.1: Authors should provide information on how to access any supplemental information such as the study protocol, raw data, or programming code.	2

*Reference: Benchimol, E. I., Smeeth, L., Guttmann, A., Harron, K., Moher, D., Petersen, I., ... & RECORD Working Committee. (2015). The REporting of studies Conducted using Observational Routinely-collected health Data (RECORD) statement. PLoS Med, 12(10), e1001885.

*Checklist is protected under Creative Commons Attribution (CC BY) license.

Supplement 2: Re-categorisations

Referral source

CPFT	SLaM	Category used
• GP<br>• PRISM (Primary Integrated Service for Mental Health)<br>• Other primary health care	• General Medical Practitioner	Primary care
• A&E ED<br>• A&E department (MH)<br>• A&E referral (acute)<br>• First response service	• Liaison A&E King's College Hospital<br>• A&E Department<br>• CAMHS Kings Emergency<br>• CAMHS Lewisham Emergency<br>• CAMHS St Thomas Emergency<br>• Child and Adolescent Crisis Care Service (Croydon)<br>• Lewisham CAMHS Crisis Team<br>• Liaison A&E Croydon University Hospital<br>• Liaison A&E Lewisham Hospital<br>• Liaison A&E St Thomas' Hospital	A&E / Crisis team / First response
• Education establishment / services<br>• School nurse	• Education Services<br>• School	Education setting
• Probation services (MH)	• Courts<br>• Criminal Justice Mental Health Services<br>• Lambeth Youth Offending<br>• Police<br>• Probation Service<br>• Youth Justice Service	Justice setting
• Self referral	• Carer<br>• Self	Self or carer referral
• Children and family services	• Croydon Children's Team<br>• LA Social Service<br>• Lambeth Children Looked After<br>• Lewisham Young People's Service (LYPS)<br>• Southwark Child and Family Service	Family or care services

CPFT	SLaM	Category used
• ARC<br>• Inpatient service (CAMHS)<br>• Community mental health team (CAMHS)<br>• Internal<br>• Consultant other than A&E (acute)<br>• Psychological wellbeing service<br>• Other source of referral	• CAMHS transition to AMH<br>• Child & Adolescent Neuropsychiatry Service (Croydon)<br>• Child and Adolescent Assessment and Liaison service (Kent)<br>• Child and Adolescent Community Service Lambeth<br>• Child and Adolescent CWP Programme Lewisham<br>• Child and Adolescent Mental Health Service (Croydon)<br>• Child Health<br>• Croydon ADHD<br>• Croydon Early Intervention - Schools Services<br>• Croydon Inpatient Direct Referrals<br>• Lambeth CAMHS Assessment Clinic<br>• Lambeth CAMHS Early Intervention<br>• Lambeth CAMHS Neurodevelopmental Team<br>• Lambeth GSTT<br>• Lewisham CAMHS Generic Team (Horizon)<br>• Lewisham Neuro-Developmental Team<br>• Lewisham Park East<br>• Liaison Inpatients St Thomas' Hospital<br>• N&S Acorn Lodge Children's Daycare/Outpatients<br>• N&S Anxiety Disorders<br>• N&S Bethlem Adolescent DayCare/Outpatients<br>• N&S Bethlem Adolescent Unit<br>• N&S CAMHS Anxiety and Traumatic Stress Clinic<br>• N&S CAMHS Ash Adolescent Unit<br>• N&S CAMHS Eating Disorders Clinic	Other (including other mental health services)

CPFT	SLaM	Category used
	• N&S CAMHS Lambeth Healthy Weight Project<br>• N&S CAMHS Neuropsychology<br>• N&S CAMHS Paediatric Liaison (Kings College Hospital)<br>• N&S CAMHS SLP Bed Management<br>• N&S Forensic & STOP Team<br>• N&S OCD (Obsessive Compulsive Disorder Team)<br>• N&S Snowsfields Adolescent Daycare/Outpatients<br>• N&S Snowsfields Adolescent Unit<br>• NHS Direct Telephone or Electronic Access Service<br>• OASIS Lewisham<br>• Other<br>• Other Clinical Speciality<br>• Other MH Trust<br>• Place of Safety<br>• Southwark Adolescent Services<br>• Southwark Bed Management Referral<br>• Southwark CAMHS Early Help Service<br>• Southwark CAMHS FFT<br>• Southwark CAMHS Neuro Developmental<br>• Southwark Carelink<br>• Southwark Inpatient Direct Referrals<br>• Southwark TaMHS<br>• N&S CAMHS Paediatric Liaison (Guy’s and St Thomas’ Hospital)	

Referral urgency

CPFT	SLaM	Category used
• Routine	• Normal<br>• Within 28 days	Routine
• Urgent<br>• 24 hour<br>• Emergency<br>• 5 days	• Urgent<br>• Within 24 hours<br>• Within 7 days	Urgent / Priority

Speciality

CPFT	SLaM	Category used
• Children<br>• CASUS	• CAMH	Child
• Adult<br>• Psychological medicine	• AMH	Adult / other

Area description

CPFT	SLaM	Category used
• Community and clinic	• Team episode	Community
• Inpatient	• Inpatient episode	Inpatient

Team (SLaM)

Team name	Team type or speciality
• CAMHS Enhanced Treatment Service<br>• Child and Adolescent Community Service Lambeth<br>• Child and Adolescent Mental Health Service (Croydon)<br>• Child and Adolescent Outreach service (Kent)<br>• Croydon CAMHS Getting Help Team<br>• Croydon CAMHS Getting More Help Team<br>• Croydon CAMHS Getting Support with Risk Team<br>• Croydon CAMHS Single Point of Access<br>• Lambeth CAMHS Assessment Clinic<br>• Lambeth CAMHS River Team<br>• Lambeth CAMHS Spring Team<br>• Lewisham CAMHS Generic Team (Horizon)<br>• Southwark Adolescent Services<br>• Southwark CAMHS Early Help Service<br>• Southwark CAMHS Outreach Team<br>• Southwark Child and Family Service	General community CAMHS
• N&S CAMHS Eating Disorders Clinic	Eating disorder or weight

Team name	Team type or speciality
• N&S CAMHS Lambeth Healthy Weight Project	
• Child & Adolescent Neuropsychiatry Service (Croydon)<br>• Lambeth Autism and Neuro Developmental Service<br>• Lambeth CAMHS Neurodevelopmental Team<br>• Lewisham CAMHS ADHD Team<br>• Lewisham Neuro-Developmental Team<br>• N&S ARD (Autism & Related Disorders Team)<br>• N&S CAMHS Mental Health Intellectual Disabilities Service<br>• N&S CAMHS Neuropsychology<br>• N&S Neuropsychiatry Clinic<br>• Southwark CAMHS Neuro Developmental	Neurological
• Child Early Intervention Service - Incredible Years (Croydon)<br>• Croydon Early Intervention - Schools Services<br>• Lambeth CAMHS Early Intervention<br>• Lewisham Young People's Service (LYPS)	Early intervention
• Child and Adolescent Assessment and Liaison service (Kent)<br>• Kings CAMHS Liaison Team<br>• Lewisham Paediatric Liaison<br>• N&S CAMHS Paediatric Liaison (Guy's and St Thomas' Hospital)<br>• N&S CAMHS Paediatric Liaison (Kings College Hospital)	Liaison psychiatry
• CAMHS Kings Emergency<br>• CAMHS St Thomas Emergency<br>• Child and Adolescent Crisis Care Service (Croydon)<br>• Child and Adolescent Crisis Care Service (Lambeth)<br>• Lambeth CAMHS Rapids Team<br>• Lewisham CAMHS Crisis Team<br>• SLaM CAMHS Response Team	Emergency or crisis team
• N&S CAMHS Anxiety and Traumatic Stress Clinic<br>• N&S CAMHS Mood Disorder Clinic<br>• N&S OCD (Obsessive Compulsive Disorder Team)<br>• Southwark CAMHS HOPE Project	Anxiety, mood or trauma
• Croydon - Looked after Children's Team<br>• Lambeth Children Looked After<br>• Lewisham CAMHS LAC Symbol Team<br>• Looked After Children's Virtual School Service (Lewisham)<br>• Southwark Carelink	Looked after children
• Croydon Youth Offending<br>• Lambeth Youth Offending<br>• Lewisham ARTS<br>• N&S CAMHS Forensic Psychology<br>• N&S Forensic & STOP Team<br>• Southwark CAMHS Youth Offending Service	Forensic or offending
• CAMHS EHCP Assessment	Other

Team name	Team type or speciality
• Child and Adolescent CWP Programme Lambeth<br>• Child and Adolescent CWP Programme Lewisham<br>• Croydon Children's Team<br>• Lewisham CAMHS Tier 4/Consultation only Team<br>• Lewisham Functional Family Therapy Team<br>• Lewisham Park East<br>• N&S Acorn Lodge Children's Daycare/Outpatients<br>• N&S CAFT (Conduct Adoption Fostering Team)<br>• N&S CAMHS CIPP Team<br>• N&S CAMHS Dialectical Behaviour Therapy<br>• N&S CAMHS SLP Bed Management<br>• N&S Oak Adolescent Daycare<br>• SEaDS<br>• Southwark CAMHS CWP	

Discharge reason

CRATE	CRIS	Category used
• Treatment completed	• On professional advice or with clinical consent	Care ended
• Transferred to other health care provider not medium/high secure<br>• Removed – procedure provided by other provider	• Internal transfer<br>• Redirected – no engagement	Care from elsewhere
• Discharged against professional advice<br>• Moved out of area<br>• Patient requested<br>• Removed / attend no treatment<br>• No reply to review letter<br>• Patient died	• Against professional advice<br>• Discharge by relative or advocate<br>• Non-attendance<br>• Death	Withdrawal or death
• Dependent caseload entry closed[a]<br>• Other	N/A	Other

[a] Mental health referrals into CPFT frequently result in multiple teams/pathways being engaged with single patients. The discharge reason "dependent caseload entry closed" implies that one of these multiple teams/pathways may have been leading care for the patient, subsequently discharged the case, and their case notes confirmed other referrals related to the patient could also be discharged.

Therapy type (SLaM)

Therapy type	Category used
• Cognitive behavioural therapy<br>• DBT	CBT or DBT
• Arts therapies	Arts therapies
• Group therapy	Group therapy
• Behavioural interventions<br>• Eclectic/integrative approaches<br>• Psychoanalytically based therapies<br>• Supportive and person centred counselling<br>• Family interventions<br>• Systems based therapies	Other

Supplement 3: Psychotropic medication names searched for in the drug variable output from NLP, and category used. Note that the coding process for identifying these names was sensitive to spelling, so relevant medications weren't captured if not spelled as written.

Medication name	Category
Agomelatine	Antidepressant
Allegron	
Alventa XL	
Amitriptyline	
Amphero XL	
Brintellix	
Cipralex	
Cipramil	
Citalopram	
Clomipramine	
Cymbalta	
Depefex XL	
Dosulepin	
Doxepin	
Duloxetine	
Edronax	
Efexor XL	
Escitalopram	
Faverin	
Fluoxetine	
Fluvoxamine	
Foraven XL	
Imipramine	
Isocarboxazid	
Lofepramine	
Lomont	
Lustral	
Majoven XL	
Manerix	
Mianserin	
Mirtazapine	
Moclobemide	
Molipaxin	
Nardil	
Nortriptyline	
Olena	
Oxactin	
Parnate	
Paroxetine	
Phenelzine	
Politid XL	

Medication name	Category
Prothiaden	
Prozac	
Prozep	
Reboxetine	
Seroxat	
Sertraline	
Sinepin	
Sunveniz XL	
Surmontil	
Tranylcypromine	
Trazodone	
Trimipramine	
Valdoxan	
Venaxx XL	
Venlablue XL	
Venladex XL	
Venlafaxine	
Venlalic XL	
Venlasoz XL	
Vensir XL	
Venzip XL	
ViePax	
Vortioxetine	
Zispin	
Abilify	Antipsychotic
Abilify Maintena	
Alaquet	
Amisulpride	
Anquil	
Aripiprazole	
Arpoya	
Asenapine	
Atrolak	
Benperidol	
Biquelle	
Brancico	
Cariprazine	
Chloractil	
Clopixol	
Clopixol Acuphase	
Clopizol Concentrate	
Clozapine	
Clozaril	
Denzapine	

Medication name	Category
Depixol	
Dolmatil	
Ebesque	
Fluanxol	
Flupentixol	
Flupentixol decanoate	
Fluphenazine decanoate	
Haldol	
Haldol decanoate	
Halkid	
Haloperidol	
Haloperidol decanoate	
Invega	
Largactil	
Latuda	
Levomepromazine	
Lurasidone	
Mintreleg	
Modecate	
Neulactil	
Nozinan	
Olanzapine	
Olanzapine pamoate monohydrate	
Orap	
Paliperidone	
Paliperidone palmitate	
Periciazine	
Pimozide	
Prochlorperazine	
Promazine	
Psytoxil	
Quetiapine	
Reaglia	
Risperdal	
Risperdal Consta	
Risperidone	
Seroquel	
Solian	
Stelazine	
Stemetil	
Sulpiride	
Sulpor	
Sycrest	

Medication name	Category
Tenprolide	
Trevicta	
trifluoperazine	
Xeplion	
Zalasta	
Zaluron	
Zaponex	
Zuclopenthixol	
Zuclopenthixol decanoate	
Zuclopenthixol dihydrochloride	
ZypAdhera	
Zyprexa	
Camcolit	Lithium and other mood stabilisers
Carbagen	
Carbamazepine	
Depakote	
Epilim	
Lamictal	
Lamotrigine	
Li-liquid	
Liskonum	
Lithium carbonate	
Lithium citrate	
Priadel	
Tegretol	
Valproate	
Alprazolam	Sleeping pills and minor tranquillisers
Alzain	
Ativan	
Axalid	
Buspirone	
Chloral hydrate	
Chlordiazepoxide	
Chlormethiazole	
Chlorpromazine	
Circadin	
Clomethiazole	
Cloral betaine	
Diazemuls	
Diazepam	
Diazepam Rectubes	
Diphenhydramine	
Dormagen	
Heminevrin	

Medication name	Category
Lecaent	
Librium	
Loprazolam	
Lorazepam	
Lormetazepam	
Lyrica	
Melatonin	
Mogadon	
Nitrazepam	
Oxazepam	
Phenergan	
Pregabalin	
Promethazine	
Slenyto	
Sominex	
Stesolid	
Stilnoct	
Syncrodin	
Temazepam	
Tensium	
Tropium	
Valium	
Welldorm	
Xanax	
Zimovane	
Zolpidem	
Zopiclone	
